# Beyond choice: social realities shaping pregnancy decisions and family planning agency among Black women in the south

**DOI:** 10.3389/fpubh.2026.1799483

**Published:** 2026-04-28

**Authors:** Terri-Ann Thompson, Yves-Yvette Evans, Monica Simpson, Oriaku Njoku Rivera, Stephanie Baker

**Affiliations:** 1 Ibis Reproductive Health, Cambridge, MA, United States; 2 Ibis Reproductive Health, Oakland, CA, United States; 3 SisterSong Women of Color Reproductive Justice Collective, Atlanta, GA, United States; 4 Access Reproductive Care-Southeast, Atlanta, GA, United States; 5 Department of Public Health Studies, Elon University, Elon, NC, United States

**Keywords:** Black women, discrimination, family planning (FP), pregnancy decision-making, pregnancy intention, reproductive justice, south, United States

## Abstract

**Introduction:**

While data on the factors associated with the sexual and reproductive health of Black women is growing, few studies have applied a reproductive justice framework to their analyses, and few have assessed the role social realities play on reproductive decision-making.

**Methods:**

We analyzed data collected from a community-based participatory study, conducted between May 2019 and January 2020. The parent study aimed to get an understanding of the reproductive health experiences and concerns of Black women living in two southern states, Georgia and North Carolina. For this paper, we used a thematic analysis to identify themes from codes within the categories: healthcare utilization, pregnancy, and family life. We applied a reproductive justice framework lens to assess pregnancy intentions, pregnancy decision-making, and family planning agency across the full spectrum of family planning.

**Results:**

In total, six focus group discussions, with 8–10 participants each, and 25 in-depth interviews were completed. Participants ranged in age, economic, and educational background. We found that social and cultural factors played an important role in pregnancy intentions and decision-making. Community and social norms worked to diminish positive feelings around Black pregnancy and non-childbearing, leading some to feel stigmatized and avoid pregnancy. Factors within the legal, economic, and health systems impacted family planning agency—limiting the ability to access desired family planning services such as abortion and infertility treatments. Participants offered strategies they believed could help counter the impact of these factors.

**Discussion:**

Our findings highlight that Black women’s pregnancy intentions, pregnancy decision-making, and family planning agency are influenced by their social realities.

## Introduction

Factors that influence sexual and reproductive health and rights (SRHR) are many, interrelated, and operate at various levels (1). Researchers have demonstrated that factors such as the economic status, residence, agency, sociocultural and gender norms, and the affordability, accessibility, and acceptability of reproductive health care services influences sexual and reproductive health decision-making, experiences, service use, and outcomes (1–3). Furthermore, from the psychology literature, cognitive factors such as past experience and biases (4, 5) have emerged as important influences on decision-making. As an example, medical mistrust on the part of the patient, resulting from an experience of poor-quality healthcare and/or the expectation of poor treatment by healthcare professionals has led to lower engagement with medical services among Black women in the United States (US) (6).

Evidence on the factors that influence the sexual and reproductive experience of Black women is growing (7–9) but few studies have applied a reproductive justice framework to their analyses (10) and few have assessed how social realities might constrain or influence Black women’s decision-making and family planning agency (11). Understanding the social reality is especially important as Black women in the United States, navigate a complex array of structural barriers related to their race and gender. Black women experience the highest rates of poverty (12), have lower access to comprehensive sexual health information (13), lower access to reproductive healthcare services (14), and have a well-established history of poor sexual and reproductive health experiences (15).

Many of the structural barriers shaping Black women’s lives are embedded in the environments where they reside. Nearly 60 % of the Black population in the United States resides in southern states (Alabama, Arkansas, Delaware, Florida, Georgia, Kentucky, Louisiana, Maryland, Mississippi, North Carolina, Oklahoma, South Carolina, Tennessee, Texas, Virginia, West Virginia, and District of Columbia) (16)—a region considered a hostile reproductive health environment. The south contains the highest concentration of counties without a single obstetrician or birthing center (17), faces severe shortages in obstetricians and gynecologists (18), requires their sex education programs stress abstinence (19), and enforces some of the most restrictive abortion policies, including total or near total abortion bans, prohibitions on telemedicine for abortion, and six-week gestational limits (20). Compounding these barriers, many southern states have not expanded public health insurance under the Affordable Care Act, leaving many low-income populations uninsured and facing high out-of-pocket costs for contraception (21)

At the same time, the legacy of redlining and chronic underinvestment in schools in predominantly Black neighborhoods has produced enduring inequities in education, employment, and economic security (22, 23). These structural disadvantages directly undermine factors—such as higher educational attainment and stable employment—that research identifies as critical to women’s autonomy in healthcare decision-making (24).

Aside from structural barriers, Black women in the United States have been subject to decades of negative portrayals of Black motherhood. Harmful rhetoric of Black mothers as “welfare queens” (25) and “crack mothers” (26), individuals who intentionally have children and misuse or abuse social systems, led to Black mothers being viewed as a drain on society and as unfit parents (26, 27). While anti-abortion campaigns stating that “the most dangerous place for an African American is in the womb” weaponized abortion use within the Black community (28). The impact of these narratives lives on, as Black women are consistently subjected to surveillance and scrutiny (29, 30) and uniquely discriminated against (31). As a result, Black women face a constrained landscape for reproductive decision-making and agency, shaped not by individual choice but by the cumulative weight of systematic inequality and discrimination.

In this paper, we use qualitative data collected from Black women living in two southern states—Georgia and North Carolina—two states with a large concentration of Black women and a mix of supportive and restrictive reproductive health policies. We aim to identify factors that influence pregnancy decision-making and family planning agency. We use a reproductive justice lens (32) to make connections between these factors and the social context as well as to showcase family planning across the full spectrum, from no children to adoption. Finally, we leverage the community-based participatory framework study approach to bring forward relevant and targeted community strategies to enhance Black women’s reproductive decision-making and agency.

## Methods

We use narratives from a qualitative community-based participatory research study that aimed to understand the (1) reproductive health experiences and concerns of Black women living in Georgia (GA) and North Carolina (NC), (2) the range of factors influencing Black women’s reproductive health decision-making, and (3) strategies that could engage and/or improve Black women’s reproductive health experience.

Black women from a reproductive justice organization and a nonprofit research organization (*the study team*) collaborated with two Black-led community organizations and a Black Research Board to conduct the parent study. Additional details on the study approach are documented elsewhere (33).

All members of the study (study team, community organizations, and research board) identify as women who have sought sexual and reproductive health services. We focused on geographic settings with a larger concentration of Black women (15% and higher) and with different reproductive health environments. Approximately 15 states have a proportion of Black women that is 15% or higher. From within these states, we selected two with a similar mix of supportive and restrictive reproductive health policies (e.g., Medicaid expansion, abortion restrictions, contraceptive equity law, etc.) (34) and where the reproductive justice organization had partners who could support study implementation.

The community organizations recruited participants for and conducted focus group discussions with 8–10 participants each. Focus group size was based on literature recommending 6 to 10 participants (35) and participant availability during scheduling. The study team trained community organizations in conducting focus-group discussions before the study was launched. Participants from the focus group discussions who wished to share more about their reproductive health experience were invited to complete an in-depth interview with a member of the study team. Data was collected between May 2019 and January 2020.

To participate in the study, people had to be between the ages 18–49, speak English, self-identify as Black or African American, and live in Georgia or North Carolina for at least 2 years. Participants with a range of educational and economic backgrounds were recruited from urban and suburban centers, through social media, flyers distributed at health clinics, other community organizations, and other spaces where Black women in the community could be reached. Interested participants were screened by telephone to assess their eligibility to participate in the study. To allow the community partner to schedule focus group discussions at a time that works for participants as well as create age-specific focus group discussions, eligible participants were asked to complete a short demographic survey over the phone and to provide days and times when they could participate in a focus group discussion. Recruitment activities were the same in each state. Focus group discussions were divided into three age groups; two younger (18–24), one older (25–49); and three mixed (18–49). In-depth interviews were conducted with women across the age groups. Recruitment concluded when the study team determined, through transcript review, that we had met thematic saturation.

Focus group discussions covered topics related to sex education, pregnancy, family life, and preventive sexual health care (e.g., cervical cancer screening), while the in-depth interviews focused on exploring healthcare decision-making. These topics were developed based on a review of the sexual and reproductive health literature and refined through conversation with the Research Board. We used a photo-elicitation methodology (36) to prompt responses to each topic. As an illustration, focus group participants were shown photos of Black families (e.g., same sex, single parent, and heterosexual) and asked, “What comes to mind when you see this image?”. Based on the prompt, participants freely generated topics. Community partners conducting the focus group discussion were trained to probe each topic raised. Illustrative probes include: “can you say more about the topic you raised?” “what are some of the challenges associated with the topic you raised?”, “what would you say are the needs of Black women around this topic?”, and “are there other concerns related to this topic that have not been raised?”. Participants were allowed to build on each topic raised and to offer their personal and/or community observations.

For in-depth interviews, participants were asked questions about their recent and past reproductive health experiences. Interviews began with questions about their general health and then more specifically about their reproductive health. They were asked to elaborate on their reproductive health needs, any concerns they have with their reproductive health, and experiences with specific reproductive health issues such as pregnancy, miscarriage, sexually transmitted infections, etc.

Notably, participants referred to themselves and those they described as women. While our results mirror the language of the participants, we acknowledge that trans, non-binary, and gender expansive Black individuals may be similarly or differently influenced by the factors that emerged in this study. Additionally, while several participants revealed that they were in same sex relationships during discussions, only a few participants discussed family formation outside of heterosexual contexts.

Focus group discussions and interviews were audio-recorded, transcribed, and quality checked for accuracy. Study participants received $50 for participating in a focus group discussion and an additional $25 if they completed an in-depth interview. The study was reviewed and approved by an independent ethics review board, the Allendale Institutional Review Board, TBW 012018.

### Analysis

For the parent study, the research team identified initial codes, developed a coding index, and assigned codes to the categories in the coding index. One interview was excluded from analysis based on concerns for data integrity. The study team developed a codebook based on the semi-structured interview guide and evidence from the sexual and reproductive health literature. One focus group transcript and two interview transcripts were independently coded by two members of the study team (YY and TT). Code discrepancies were reconciled by the study team. To ensure codes remained consistent with their definitions and across reviewers, the study team reviewed them at multiple time points, including before they were shared with community reviewers (*research board and community organizations*). One study team member maintained an audit trail capturing decisions from code reviews, interpretation, and theme discussions. The audit trail also included notes taken during in-depth interviews and debriefs following focus group discussions. Codes and code categories were also reviewed carefully during thematic analysis. Initial codes were combined to form larger categories.

All members (*the study team, research board, and community organizations*) reviewed, discussed, and approved the codes and categories that emerged from the analysis. No code definitions or categories were changed during community review. Community reviewers agreed with the interpretations of the data and discussed in detail how to minimize unintended harm to the community.

Two study team members (TT and YY) have significant experience collecting and analyzing qualitative data related to sexual and reproductive health, including training in and experience conducting interviews.

For this paper, we used a thematic analysis (37) to identify themes from codes within the categories: healthcare utilization, pregnancy, and family life. We began by identifying the full range of sexual and reproductive health experiences such as contraception, birthing, postpartum care, abortion, etc. captured across the three categories. We deductively derived codes from each of these experiences related to reproductive justice principles: ‘the right to have a child’ and ‘the right to not have a child’. For this study, these principles were operationalized as pregnancy intention (desires to become pregnant), pregnancy decision-making (evaluating options related to pregnancy), and family planning agency (ability to decide and attain desired family planning services). We refined these codes inductively to create themes that highlighted various influences on pregnancy intention, pregnancy decision-making, and family planning agency. Because the reproductive justice framework is inherently intersectional, it acknowledges that influential factors operate across multiple socio-ecological levels and may work in concert or independently. When more than one factor could explain a theme, we organized our findings based on which factor best reflected power, structural conditions, or an inequity for the specific reproductive health action (pregnancy decision-making, family planning agency, or pregnancy intention). As an illustration, economic restraints were most relevant to the theme that reflected structural barriers that limit family planning agency.

Across the themes, we looked for patterns that emerged based on the participant’s age/age group, education, insurance status, and state. Themes were refined based on the research aims. The qualitative data was managed using the software program Dedoose (38).

Excerpts from focus group discussions and interviews are represented by state and participant age/age group in the case of focus group discussions.

## Results

In total, 49 women participated in the study: 20 from North Carolina and 29 from Georgia. Six focus group discussions, 4 in Georgia and 2 in North Carolina were conducted, and a subset of participants (*n* = 14 from Georgia and *n* = 11 from North-Carolina) completed an in-depth interview. On average, focus group discussions lasted 122 min, while in-depth interviews averaged 48 min. Participants ranged in education, from high school or below to masters/doctorate. However, nearly half of the participants held a bachelor’s degree. Most participants reported being employed full time. A third of the participants had state-funded health insurance coverage and close to 50% had insurance through a private entity, employer, or school. They ranged in age, from 18 to 47, but the median age in both states was 27 years. Not all participants reported on their pregnancy history (*n* = 3), but of those who did, 55% had experienced pregnancy (see Table 1).

**Table 1:** Participant characteristics.

**Characteristics**	**Number (*n* = 49)**	**Percent (%)**
State		
Georgia	29	59
North-Carolina	20	41
Age		
Median (range), years	27 (18–47)	
Education		
High school or below	11	22
Associates/some college/trade	6	12
Bachelor’s degree	20	41
Masters/doctorate	9	18
No response	3	6
Employment		
Full time	28	57
Part-time	15	31
Unemployed	5	10
No response	1	2
Insurance status		
Public, or other	16	33
Employer, school, or private	23	47
No insurance	4	8
No response	6	12
Pregnancy history		
Ever pregnant	27	55
Never pregnant	19	39
No response	3	6

Three themes emerged from the analysis. Themes highlight influences on pregnancy intention, decision-making, and family planning agency. Social and cultural factors were named as influencers for pregnancy intention and decision-making while health, legal, and economic factors influenced family planning agency. The final theme brings to the fore, personal and community strategies that could improve decision-making and agency among Black women. No differences in themes were noted by state, education, or insurance status. However, we did observe differences by age/age group for the social and cultural theme, with younger participants citing a greater negative influence of social and cultural factors on pregnancy intentions and decision-making. Findings are presented by theme.

### Social and cultural factors influence pregnancy intentions and decision-making

Narratives from both focus group discussions and in-depth interviews highlight several ways in which the social context influenced Black pregnancy intentions and decision-making. Within their social and cultural environment, participants named norms at the community and societal level and experiences of discrimination as influential.

First, single parenthood was named as both a community and societal norm that influenced pregnancy decision-making. Single parenting was frequently observed within their communities and commonly perceived at a societal level as the norm for Black women. One commonly cited source for this perception was media portrayals of Black women: *“[…] it’s a propaganda scandal, […] no matter if the woman has a man or not, that’s the first look that their [sic] media is going to put out there is that every Black woman is single and pregnant, and that’s what they’re looking at us (Black women) as”. ~Georgia, 18–49.*

These norms were viewed negatively by participants. Participants acknowledged that single parenting translated into less involvement of male partners in childcare, more responsibility for child bearers, economic hardship, and negative feelings about pregnancy. Three participants described pregnancy as a “negative thing” for Black women based on their observation that it did not often occur within the context of a secure relationship or stable finances. This negative perception resulted in some, particularly younger participants, reporting no desire to experience pregnancy, while others imposed economic stability (in the form of greater finances or partnership) as a requirement for pregnancy and a constraining factor for family size.

*“I think of this myself, […] I just never want kids, […] – it’s sort of like an ending. It’s definitely a negative thing for Black women, where (whereas) if you get pregnant there’s like a larger sense of security or you may be in a more secure relationship or benefits conferred to you and all that stuff for the white women and upper-class women.” ~Georgia, 18–24.*

In tandem with the single parenthood norm, participants reported that pro-childbearing norms within the community led some Black women to refrain from expressing their desire for no children, to feel stigmatized when they expressed a desire for no children, to repeatedly have to affirm their desires for no children, and to feeling isolated. To avoid this judgement, some avoid seeking the services that align with their family planning desires such as abortion services.

*“I think when it comes to women who don’t want children or who, when they’ve become pregnant, they regret that decision, we should get rid of the stigma and get rid of the shame of like (a) not wanting to be a mother, and (b) abortion, […].” ~Georgia, 18–49*

Community norms of pro-childbearing conflicted with perceptions that the society viewed Black pregnancies as undesired and devalued.

*“Like really if you’re a minority, […] Why are you getting pregnant? It’s, like a stigma. […]White women have kids all the time. Asian women have kids all the time.” ~North Carolina, 37*

The feeling that Black pregnancies were not welcomed within society was disconcerting and created a sense of confusion when paired with community pro-childbearing norms. Specifically, participants named that pregnancy, a desired community outcome, felt more subdued and overly complicated rather than celebrated for Black women. Further, the dissonance created by the perception that black women’s pregnancies were not desired had implications. A younger participant reflected on how perceptions that Black pregnancy was devalued may subconsciously manifest into fewer Black women pursuing fertility options.

*“I think that a lot of reason why we don’t see it (infertility treatments) in Black women as much is because […], for Black women you see more people talking about getting their tubes tied or getting all these other things to prevent pregnancy. You don’t usually hear us (Black women) wanting to – well, you don’t usually hear the media or anybody else talking about Black women wanting to get pregnant, it’s more so they’re trying to stop us from getting pregnant.” ~Georgia, 18–24*

Finally, personal experiences, along with stories shared within families and across communities about the poor care and treatment Black women receive during pregnancy and delivery, as well as news reports of high maternal mortality rates in the states, influenced pregnancy intentions. This was especially true for younger participants. Younger participants named that their negative and the negative experience of other Black women within medical institutions such as hospitals and clinics diminished their desires to get pregnant.

*“One reason I don’t want to experience pregnancy is like there’s just so much that can go wrong, it seems like. […] So it’s sort of hard to wrap my head around the idea that someone can be your doctor and not be invested in you living or in whatever pain that you might be experiencing that may like – while you’re delivering a baby it’s certainly something to pay attention to. It’s hard to wrap my head around someone allowing me to die while I’m trying to – or I’m delivering a baby, so that’s a big fear.” ~Georgia, 18–24*

The impact of experiences and stories of medical discrimination were compounded by concerns about building families in a discriminatory society. Narratives highlighted heightened surveillance of Black people, an unfair judicial system, and violence against Black men as concerns. Participants reported that these concerns were present when contemplating pregnancy (i.e., starting a family) and continued to surface throughout pregnancy, childbearing, and childrearing. The impact of this concern on pregnancy intention, specifically diminishing desires for pregnancy, was raised across age groups.

### Health systems, economic factors, and policies influence family planning agency

Moving beyond pregnancy intentions and decision-making, participants highlighted that health systems, economic factors, and policies influenced their family planning agency.

Beginning with the health system. Participants described how past interactions with healthcare providers diminished their sense of autonomy and impacted their ability to make informed choices. Across multiple reproductive healthcare services (contraception, pregnancy, birthing, and postpartum care), participants related a consistent feeling of being dismissed by providers and mistrusting the medical advice and treatment that was offered. Illustrations of this concern surfaced when participants discussed the high prevalence and poor treatment of reproductive conditions such as fibroids and endometriosis within the Black community. In both cases, participants shared that discussions centered treatments that reduced their reproductive prospects, such as hysterectomies.

“*A lot of Black women suffer from endometriosis and no one really talks about it. Nor do we discuss fertility options for those individuals. It’s just kind of, you’ve been relegated to a life of” you probably will not be able to have kids “and then that’s just it. Nobody discusses family planning or how to get around it, cause it is still possible to have children. It’s just that nobody’s instilling that type of hope*.” *~North Carolina, 18–24*

Further, conversations with providers about ways to prevent pregnancy felt limited and/or made them feel like their preferences would not be honored or taken into consideration. As one participant shares:

*“I think she (the gynecologist) would, […] be receptive to hearing what I’m saying. But she’s pretty much, I feel going to have her mind made up already. So, she may listen to me but then come back and say, “Okay, so here’s what we are going to do.“Not really taking into account anything that I’ve just said. I guess it was just something that we (Black women) think is normal. We just assume people aren’t going to listen to us so we just go with it.” ~North Carolina, 38.*

Participants expressed wanting greater details about contraceptive methods from their healthcare providers, particularly more transparency and discussion about the side effects as well as inquiries into whether and what side effects would be most acceptable. Many reflected that while they wanted to avoid pregnancy, the side effects from birth control pills and/or poor experience with an IUD led them to discontinue the methods. Many remained without a contraceptive method because they did not return to a provider to discuss alternatives and/or assumed there were no alternatives.

*“I think, just maybe being able to more extensively talk about, you know, birth control methods. […] I would like a form of birth control that was not hormonal, but also wasn’t an IUD… and like wasn’t a condom. […] I wish I had a form of birth control that I felt like I could use without experiencing great discomfort or inconvenience” ~Georgia, 35.*

Economics, specifically fewer financial resources and insurance coverage were named as a barrier and facilitator, respectively to pursuing family planning services, thereby influencing agency. Across participants, insurance coverage was described as essential to accessing family planning services, allowing women to avoid or delay having children. Participants with insurance coverage were able to obtain pregnancy care, abortion services, and delivery care. However, participants also recognized the limitations of insurance coverage including that some family planning services were not covered or were only partially covered.

*“I just recently graduated from Georgia State as a grad student. […] I paid insurance through the school so I didn’t have to think about it [family planning services].[…] I would go to Georgia State and get that taken care of.” ~ Georgia, 18–49*

Participants with state-funded health insurance and no insurance were especially attuned to the prevalence of people in their community with state-funded health insurance, who go without insurance for long stretches of time, or have no insurance at all. Discussions about health insurance were often tied to employment. They highlighted that many of the jobs available to them and others in the community were hourly, low paying, offered no insurance benefits, and that obtaining insurance in these circumstances could further strain household finances.

*[…] my situation is very common. I’m getting paid $10 an hour until your employer’s like, “Oh, you just got a raise,” and it’s like, “Oh, with this raise comes insurance, Oh that’s a beautiful thing.” So now my $10 an hour, I’m making, okay, I’m doing 40 h a week; that’s $400. You’re taking another 150 to $200 out of my check every month to go towards insurance and I still have to pay rent; I still have electricity; I still have a kid to feed on top of everything else. So, then its like now you are faced with the fact, do I take the insurance or do I live?” ~ North Carolina, 18–49.*

Obtaining health insurance outside of employment was not considered feasible, neither was paying out-of-pocket for family planning services—even at clinics typically perceived as more affordable. In the absence of insurance coverage, participants described having to travel beyond their neighborhoods, sometimes over long distances, to locate health centers or providers that offered services for free or on a sliding scale basis.

*“Two years ago, I was using contraceptives sporadically and just hoping I wasn’t […] contracting an STD. […] Two years ago, I had no health insurance, so I had to rely on like free-standing clinics.” ~Georgia, 27*

*“I had no insurance […] and so, I paid out of pocket for all of my care, and they charged me $70 to take out the IUD. And I had to go really far to even have them do a sliding scale for me to be able to take out my device” ~Georgia, 18–49*

Without insurance coverage, some participants were forced into precarious situations. In one narrative, a participant with no insurance at the time of her pregnancy, recounts being forced to carry a pregnancy to term after visiting a crisis pregnancy center for a free pregnancy test to date the pregnancy.

*“[…] So my initial thought was not to carry, to term, my pregnancy. It was to terminate my pregnancy. At first, my first resource was abortion clinic. It wasn’t… It wasn’t to go through with my pregnancy, but I went to a pregnancy crisis center that I didn’t know that were very religious based. I went in on like, “Oh, we do free pregnancy tests, and then we help you with abortion. So I went there, found out I was pregnant, but instead of going with my wishes, they gave me an ultrasound and let me hear the baby’s heartbeat. Then it was like, “They want you to keep the baby. They’ll help you. Blah blah blah.” That’s a lie. As soon as you get past like 4 months, you do not hear from them anymore. If you need any services, you do not get any services, they’ll just point you, “Oh this is DSS or this is where you get this, that and then a third. […]” ~North Carolina, 24.*

Participants across employment and insurance statuses described financial constraints as a common challenge within Black communities, noting that these constraints sometimes resulted in limited or no engagement with certain family planning services. This finding first surfaced in relationship to insurance coverage. Several participants noted that even with coverage, they had to forgo utilizing health services because co-payments presented a challenge. During discussions around infertility, abortion, and contraception needs, multiple participants highlighted their inability to seek out desired services because they did not have the financial resources. Participants highlighted the high cost of infertility treatments (a service that could help them to start a family) were beyond their and other Black women’s reach. Further, for those who did not want to have children, an inability to cover the cost of abortion and contraceptive methods such as sterilization were also highlighted.

*“There was a period of time where I was trying to get pregnant with my boyfriend at the time. It just, it wasn’t happening, and we couldn’t understand why. I wanted to go and take a fertility test, go see a fertility doctor. The price of that is outlandish. There was no way possible.” ~North Carolina, 18–24.*

For those not wanting to have children at a specific moment in time or ever, policies made it difficult to access or engage with desired family planning services such as abortion. Many participants discussed abortion bans in the larger context of reproductive health, citing (1) the hypocrisy of politicians, as welfare funds are limited and frequently debated, (2) the discriminatory impact of the laws on Black women, and (3) a cynicism about the promises of politicians to prioritize Black women’s health.

*“[…] you’re damned if you do, damned if you don’t […] you can’t have access to equitable healthcare for your baby, but you also don’t have an option and you are forced to have the birth, as well […] It’s connected to not really taking care of Black women and making sure that Black women are getting access, and to keep Black women from getting equitable access to the things they need.” ~Georgia, 27.*

Participants, especially those in the Georgia focus group discussions, were attuned to legislative proposals that restricted abortion access. At the time of the study, the governor of Georgia had introduced a bill to prohibit abortion services except in certain situations, after the sixth week of pregnancy. The bill incited a range of emotions among participants, from anxiety to anger.

Finally, participants raised adoption as an accepted way to expand families (*an extension of family planning*) for Black women with no desire for biological conception or who face fertility struggles. While informal pathways to adoption were seen as possible and commonplace, such as aunts raising their nieces and nephews, formal adoption was described as more challenging for Black families. To adopt or foster a child, the hosting family is subject to background checks. This policy, though considered standard practice, has a discriminatory impact on Black families—working against their parenting desire. As one participant explained: *“[…] when you’re going through adoption, they have to go through everybody in the household. Unfortunately, in the African American community, we find that a lot of people and our families have been incarcerated or have some kind of charges, felonies, or anything against them. If you have a husband, a child, or anything in your household that has any type of record, that prevents you from adopting. […] for most Black people, that’s something that they have to come up against a lot.” ~North Carolina, 18–24.*

### Personal and community engagement as tools for enhanced decision-making and agency

Descriptions of the factors that influenced pregnancy decision-making and family planning agency were often paired with personal and community strategies to counter their impact. Notably, while participants raised broadly that policy changes were needed, recommendations were much more focused on what they or the collective Black community could do to improve reproductive decision-making and agency.

At the individual level, several participants named finding a Black physician as a ready tool to enhance the decision-making and agency. Black physicians were described as more empathic and less likely to dismiss family planning concerns. While it was acknowledged that this resource was limited, especially in whiter and more rural areas, participants stressed that the burden of finding and potentially traveling long distances to reach a Black physician were worthwhile.

*“It’s just like the importance of Black doctors. I know that once I got health insurance, I was like I have to have a Black female doctor. And so, I’ve been driving all over just to go to my [Black] physician.” ~Georgia, 18–49.*

At the community level, participants described the need for more space within the Black community to openly discuss pregnancy intentions and decision-making as well as challenges with conception. They believed that open spaces would offer a sense of solidarity—thereby reducing the stigma and isolation experienced by those who do not want to have children or who are struggling to have children.

*“I also think we need to start talking more up front about the Black women – especially Black women who don’t want to have families, because like I said, I think I want to be married, but I don’t – I know for a fact that I don’t want to have a child, and I know there’s a lot of women who are like me” ~Georgia, 18–49.*

Participants also emphasized the ability to use the shared space to equip Black women in their communities with more information on (1) reproductive health conditions and the options for treatment, (2) supportive health services such as doulas for pregnancy, and (3) stigmatized services such as abortion. In so doing, they believed Black women would feel more empowered to assert their family planning desires as well as act on them.

To *“prepare Black families for the society their children are being born into”*, participants suggested that the community develop a book and put together classes for Black men, women, and families on how to talk to their children about race. This information might reduce some of the anxiety around and negative feelings towards Black pregnancy.

Finally, participants suggested the community advocate for different and affirming media portrayals of Black women. They wanted to see representations of Black women seeking fertility treatments, Black women celebrating pregnancies, and broader depictions of Black families—including those without children and extended family members. Participants hoped that these portrayals would spark conversations within the Black community about the full spectrum of pregnancy intentions, moving beyond the narrow dichotomy of “childbearing now or later”. Expanding this spectrum, they suggested, could foster greater acceptance of a wide range of family planning choices and family structures, including abortion and childfree families. Across the focus group discussions, participants reasoned that broader depictions of Black families could encourage others in the community to interrogate longstanding stereotypes about Black pregnancy and single parenthood, mirroring the reflections participants arrived at during the discussion. As an illustration, participants questioned their notions about single parenthood being ‘bad’ during the focus group discussions and pushed back against the narrative, citing a diversity in Black family structures, the different roles that family members can play in raising children, and that the presence of a single biological parent does not, on its own, define a family as a single-parent household.

*“And even with the single motherhood, is single defined by having a partner? Do like polyamorous people count? Do non-married committed people count as single parents? What about non-nuclear family-based family structures where the parent might be the only parent, but they have a supportive community of like a grandmother or great-grandmother or aunty or a cousin or whatever, or several cousins? […] it just always feels like we’re [Black women] trying to meet a standard that for whatever reason isn’t working for us, and finding ourselves limiting, like not meeting that standard, when that standard isn’t necessary to be happy or successful.” ~GA, 18–49.*

## Discussion

This study offers insights on pregnancy intention, pregnancy decision-making, and family planning agency using the lived experiences and perceptions of a sample of Black women residing in two southern states. From our qualitative data, we identified social, cultural, interpersonal, and structural factors influenced pregnancy intentions and decision-making and family planning agency. Negative perceptions of Black pregnancy in addition to stigmatization of non-childbearing Black women led some to avoid pregnancy, constrain their family planning choices, and/or refrain from expressing their true family planning desires. Community stories highlighting poor pregnancy and delivery outcomes (e.g., high rates of maternal death) for Black women diminished the positive feelings related to pregnancy—ultimately leading some to want to forgo the experience. Our findings on medical bias and the resulting strategy named by Black women supports existing evidence that mistrust of the medical system informs Black women’s decision-making around pregnancy (39). It extends this evidence by highlighting that some Black women may choose to forgo pregnancy altogether because of this mistrust.

Narratives on single parenting norms, namely economic precarity and lower male involvement, mirror statements from the Denson study (11), which highlights the stress and uncertainty associated with parenting alone. However, our findings that this stress and uncertainty diminish pregnancy desire and impacts pregnancy decision-making differs from Denson, whose participants reflect a more limited impact of male partner involvement on pregnancy intention or decision-making. These differences could be due to differences in applied methodology and/or variations in the study population.

Black women in our study highlighted that living in a context where Black people are more likely to be targeted, detained, charged, and sentenced than their White counterparts (40) instilled a sense of anxiety around having children and that those anxieties arose early, when some Black women were contemplating pregnancy. While existing literature shows the impact of racism during pregnancy and birthing (41, 42), our findings add to the literature by suggesting that the apprehension around pregnancy that results from experiences of racism emerge long before pregnancy and delivery. In our study, participants recommended parenting classes and books for Black families raising their children to reduce some of the anxiety. This suggestion is in keeping with racial socialization (43), a strategy highlighted in the literature that African Americans have used to prepare their children for managing racial prejudice and discrimination. Our findings around the early timing of these anxieties suggests that racial socialization strategies such as a book for Black families could be made available earlier in the pregnancy journey (i.e., to Black women who are considering starting a family versus later in pregnancy or post-delivery). Existing research identifies racial pride as a factor that explains how “Black youth may thrive despite exposure to discrimination” (44). Programs such as ‘Strong African American Families’, (44) a culturally relevant intervention designed to buffer Black families and youth from the harmful effects of discrimination, incorporate elements of racial socialization and racial pride. This program could be used in community settings to help ease the apprehension some Black women feel around pregnancy because of racism. Finally, our participants’ recommendation for community spaces to discuss health information could be leveraged to offer peer coaching and/or general support for those considering pregnancy. Peer coaching has been successful for other maternal health issues in the Black community (45) and could be adapted for this purpose.

Wealth disparities between White and Black households in the United States are well documented. A report from the Center for American Progress found that Black women working full-time, year-round earned nearly 40% less than white males (46). Further, Black Americans are also disproportionately concentrated in low-wage jobs (47), overrepresented in state-funded health insurance programs, and face higher uninsured rates than their White counterparts (48). Finally, both North Carolina and Georgia have median household and per-person incomes that fall below the national average (49). Against this backdrop, it is no surprise that limited financial resources and gaps in health insurance coverage emerged in our study as structural factors shaping family planning agency. While health insurance can support family planning agency, policies that restrict coverage of specific reproductive health services (50), or exclude services altogether—such as infertility treatments (51)–require Black women to secure additional funds to access care. In financially constrained contexts, this often proves challenging and can result in some women being unable to obtain desired services.

No differences in themes were noted by state, likely due to similarities in reproductive health policy environments across the two states. The two southern states have abortion restrictions, lack coverage for infertility treatments, have adopted Medicaid expansion, and maintain contraceptive equity laws that do not cover all forms of contraception (34). Our findings on the influence of legal factors centered on abortion restrictions. Participants viewed these restrictions as discriminatory because they are concentrated in spaces where many Black women live and because they limit access to a service that could help women achieve their desired family size or avoid childbearing altogether. While there is evidence showing the impact of economic and legal factors on family planning (52–55), our study findings bring the needs of Black women who do not want to have children, who might seek adoption, and who are struggling with infertility into sharper focus.

Black women’s pregnancy decision-making is not immune to their social reality; a reality where Black pregnancy is negatively portrayed and seemingly unwanted and where medical advice could be limited or coercive. Other factors highlight the impact restrictions on insurance coverage and health service provision (e.g., abortion coverage restrictions and abortion bans) have on a population that is more likely to need abortion and contraception services due to disparities in the risk of unintended pregnancy (56, 57).

Factors also work singularly or collectively on pregnancy intentions and decision-making and family planning agency. As an illustration, pro-childbearing norms stigmatize infertility, abortion, and people who do not want children. Stigma around these issues leads to isolation and to some not using services such as abortion to avoid judgement. When these norms are paired with factors such as financial constraints, restricted insurance coverage, or restrictive legislation, it further reduces agency. Our findings on the isolation some Black women feel around infertility, complements other qualitative and intersectional work (58) that highlights the role social relationships, stereotypes, and norms play. We add to the literature by showing the compounding effect of factors such as finances and that fertility is an important concern for Black women.

Finally, our study offers some community generated insights into areas that, if improved, could have a positive impact on Black pregnancy intention, pregnancy decision-making, and family planning agency.

Single parenthood and pro-childbearing are among the narratives raised in our study that permeate the context within which pregnancy decision-making occurs. These narratives emerged at the individual, community and society level. Some narratives felt external and beyond their control, such as the continued negative portrayal of Black families in the media (59); while others felt more proximate and receptive to change, such as increased stories about the use of assisted reproductive technology in news and social media outlets (60). While there was acknowledgement of the consequences of these narratives—negative feelings around pregnancy, isolation, and shame—participants actively pushed back against negative narratives related to the desire to not have children as well as the reified notion of single Black parents being the sole family archetype in Black communities and called for different and more positive portrayals.

Counter-stereotypical images and narratives as well as stereotype replacement are among the ready interventions that may be used to effect reductions in bias and that would support participants’ ask for different narratives (61–63). Increasing funding for organizations that use these strategies to challenge reproductive and racial stereotypes—and that operate nationally—could broaden public exposures to healthy Black women across the life spectrum (single, mothers, widowed, etc.), to celebratory images of Black pregnancy, and to Black women engaging in a wide range of family planning choices, from preventing to delaying to starting families. Additionally, in our discussions of reproductive healthcare, participants highlighted the power of storytelling within the community. Building on this, storytelling—an approach that has been used effectively to teach African American women about reproductive health (64)—could also be employed to increase exposure to diverse narratives around pregnancy and family planning in Black communities. The goal of these exposures, inside and outside the Black community, is to support Black women’s autonomy in their sexual and reproductive lives.

To address the recommendation for more spaces where health information could be shared with Black women, services offered at community-based organizations that center both Black people and access to sexual and reproductive health care could be leveraged or increased. Community-based organizations can (1) effectively communicate health information and facilitate health promotion (65), (2) provide spaces for Black women to convene and share support, and (3) organize and harness community power to advocate for changes within larger systems (66).

### Implications

Findings from this qualitative study are hypothesis generating and offer programmatic solutions. With regards to our findings on medical bias. Future research studies could evaluate the impact of (1) policies and/or programs on cultural sensitivity training in family planning and (2) race concordance in family planning providers on Black women’s pregnancy intentions and family planning agency. Specifically, these studies could seek to understand whether feelings of autonomy, coercion, and validation are improved in cases of race concordance or following cultural sensitivity training. Separately, qualitative studies on pregnancy intentions and family planning agency with Black women living in different geographic and sociopolitical contexts may further elucidate the pathways through which the factors identified (social, cultural, economics) influence pregnancy intention, pregnancy decision-making, and family planning agency. We proposed programming that aligns with participant recommendations for enhancing pregnancy decision-making and family planning agency within the Black community. Evaluations of these programs could be conducted to assess (1) changes in sexual health knowledge, literacy, and empowerment and (2) whether these programs could be replicated across other community settings.

While this study does not point to direct policy solutions, advocates working to remove abortion restrictions inclusive of bans on abortion coverage in state-funded health insurance plans, could use these findings to highlight Black women’s interpretations of such restrictions as antithetical to politicians’ promise to prioritize Black women’s health, and to illustrate how insurance coverage limitations shape family planning agency within this community.

### Limitations

While the sample consists of Black women recruited from clinics, community spaces, and living in urban/suburban areas the experiences of Black women living in rural areas as well as Black women with higher income status is missing from our sample. Therefore, factors specific to these characteristics may be missing from our analyses. Additionally, some of the factors that emerged in our analyses may not apply or may exert influence at a much lesser degree on these groups of women. That said, we know from the literature that experiences of discrimination in the US cut across income and residential lines and that reproductive outcomes, particularly birthing outcomes, are not significantly better for Black women with higher education or higher incomes. While we believe the photo-elicitation method generated responses that were of the most importance to participants, not asking participants directly about the factors that influenced pregnancy and family planning, could have produced different, enabling, and/or additional factors than those covered in this paper. We were unable to collect pregnancy histories from all participants who engaged in a focus group discussion. Responses of the three participants without pregnancy history could not be removed from our analysis. Finally, while our findings were shared with and vetted by our community members (research board, community organizations, and reproductive justice organization), they were not shared with our participants. Member checking would have strengthened the study by ensuring that the interpretations assigned to the qualitative data accurately represent participant perspectives.

## Conclusion

Narratives from Black women in two southern states brings to light the many factors that influence pregnancy intentions, pregnancy decision-making, and family planning agency. They highlight the important role of community norms, negative societal perceptions, medical bias, along with economic and legal factors. These findings hold implications for those working to shift narratives around Black pregnancy as well as for those operating in clinical and programmatic sexual and reproductive health spaces. Future studies include qualitative research on pregnancy intentions and family planning agency with Black women in different geographic and sociopolitical contexts to further elucidate the pathways through which the identified factors (social, cultural, economics, etc.) influence pregnancy intention, pregnancy decision-making, and family planning agency.

## Data Availability

The datasets presented in this article are not readily available because participants did not grant permission for wider use of the data. Requests to access the datasets should be directed to tthompson@ibisreproductivehealth.org.
