# Correction: Beyond choice: social realities shaping pregnancy decisions and family planning agency among Black women in the south

**DOI:** 10.3389/fpubh.2026.1869007

**Published:** 2026-05-11

**Authors:** Terri-Ann Thompson, Yves-Yvette Evans, Monica Simpson, Oriaku Njoku Rivera, Stephanie Baker

**Affiliations:** 1 Ibis Reproductive Health, Cambridge, MA, United States; 2 Ibis Reproductive Health, Oakland, CA, United States; 3 SisterSong Women of Color Reproductive Justice Collective, Atlanta, GA, United States; 4 Access Reproductive Care-Southeast, Atlanta, GA, United States; 5 Department of Public Health Studies, Elon University, Elon, NC, United States

**Keywords:** Black women, discrimination, family planning (FP), pregnancy decision-making, pregnancy intention, reproductive justice, south, United States

In the abstract, text was missing from the first sentence. This has been corrected to read:

While data on the factors associated with the sexual and reproductive health of Black women is growing, few studies have applied a reproductive justice framework to their analyses, and few have assessed the role social realities play on reproductive decision-making.

The original version of this article has been updated.

